# Supplementary material for: Conventional PCR Versus Next Generation Sequencing for Diagnosis of FLT3, IDH and NPM1 Mutations in Acute Myeloid Leukemia: Results of the PETHEMA PCR-LMA Study
Source: Cancers (Basel). 2025 Mar 1;17(5):854. doi: 10.3390/cancers17050854 (PMC11898636; doi:10.3390/cancers17050854)
Supplement: Supplementary file 1 [file cancers-17-00854-s001.zip › cancers-3444725-supplementary.pdf]

Table S1. NGS and PCR results for each mutation at AML diagnosis, (NGS VAF cut-off ≥5%).

Gene mutation		PCR + N(%)	PCR – N(%)	TOTAL
NPM1	NGS +	246 (98.8)	9 (1.1)	255
	NGS -	3 (1.2)	803 (98.9)	806
	TOTAL	249 (100)	812 (100)	1061
FLT3-ITD	NGS +	124 (72.9)	3 (0.3)	127
	NGS -	46 (27.1)	906 (99.7)	952
	TOTAL	170 (100)	909 (100)	1079
FLT3-TKD	NGS +	43 (82.7)	6 (0.7)	49
	NGS -	9 (17.3)	893 (99.3)	902
	TOTAL	52 (100)	899 (100)	951
IDH1	NGS +	56 (98.2)	10 (1.4)	66
	NGS -	1 (1.8)	699 (98.6)	700
	TOTAL	57 (100)	709 (100)	766
IDH2	NGS +	92 (98.9)	18 (2.7)	110
	NGS -	1 (1.1)	660 (97.3)	661
	TOTAL	93 (100)	678 (100)	771

Abbreviations: NGS, next generation sequencing; PCR, Polymerase chain reaction; *FLT3*, FMS-like tyrosine kinase-3.

Table S2. NGS and PCR results for each mutation at R/R AML, with NGS cut-off VAF ≥5%

Gene mutation		PCR + N(%)	PCR – N(%)	TOTAL
NPM1	NGS +	16 (94.1)	1 (1.3)	17
	NGS -	1 (5.9)	77 (98.7)	78
	TOTAL	17 (100)	78 (100)	95
FLT3-ITD	NGS +	25 (78.1)	1 (0.6)	26
	NGS -	7 (21.9)	155 (99.4)	952
	TOTAL	32 (100)	156 (100)	188
FLT3-TKD	NGS +	6 (100)	2 (1.2)	8
	NGS -	0	171 (98.8)	171

	TOTAL	6 (100)	173 (100)	179
IDH1	NGS +	21 (100)	1 (0.7)	22
	NGS -	0	137 (99.3)	137
	TOTAL	21 (100)	138 (100)	159
IDH2	NGS +	15 (100)	1 (0.7)	16
	NGS -	0	144 (99.3)	144
	TOTAL	15 (100)	145 (100)	160

<u>Abbreviations</u>: NGS, next generation sequencing; PCR, Polymerase chain reaction; *FLT3*, FMS-like tyrosine kinase-3.

Figure S1. Sensitivity and specificity at diagnosis with NGS cut-off VAF ≥ 5%.

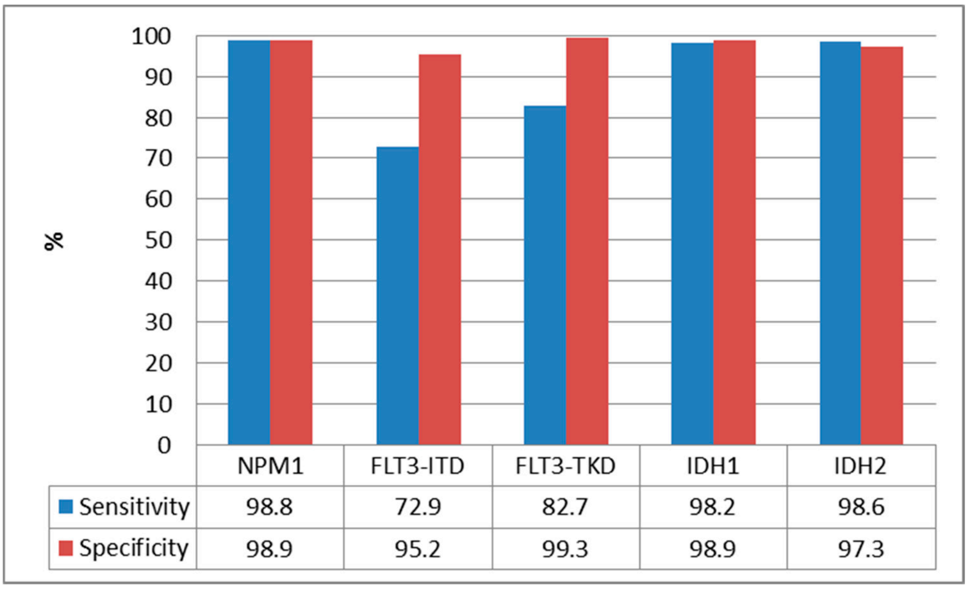

Figure S2. NGS sensitivity and specificity at R/R AML (NGS cut-off VAF ≥5%).

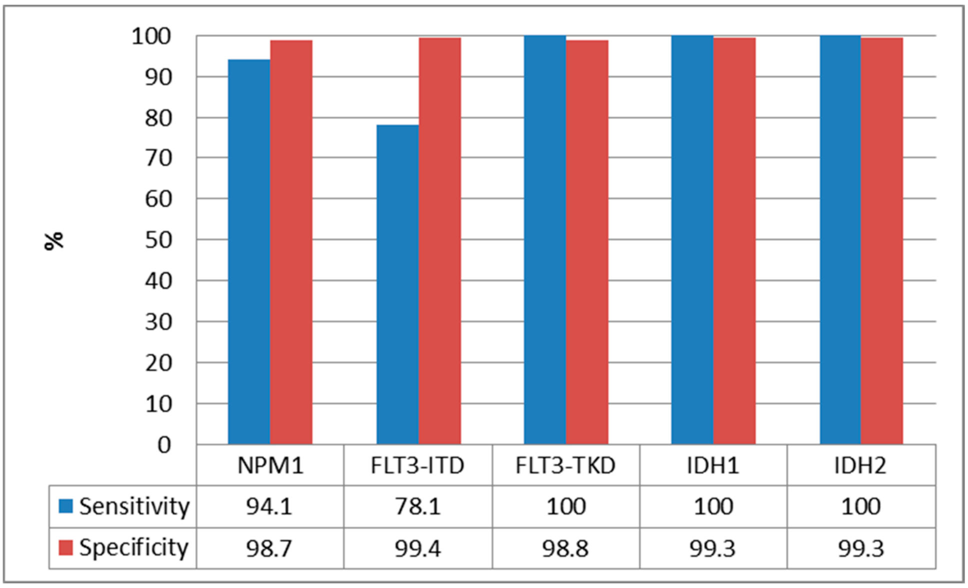

Figure S3. Number of days elapsing from specimen receipt to diagnostic report, A) overall, B) at diagnosis, and C) at R/R episode.

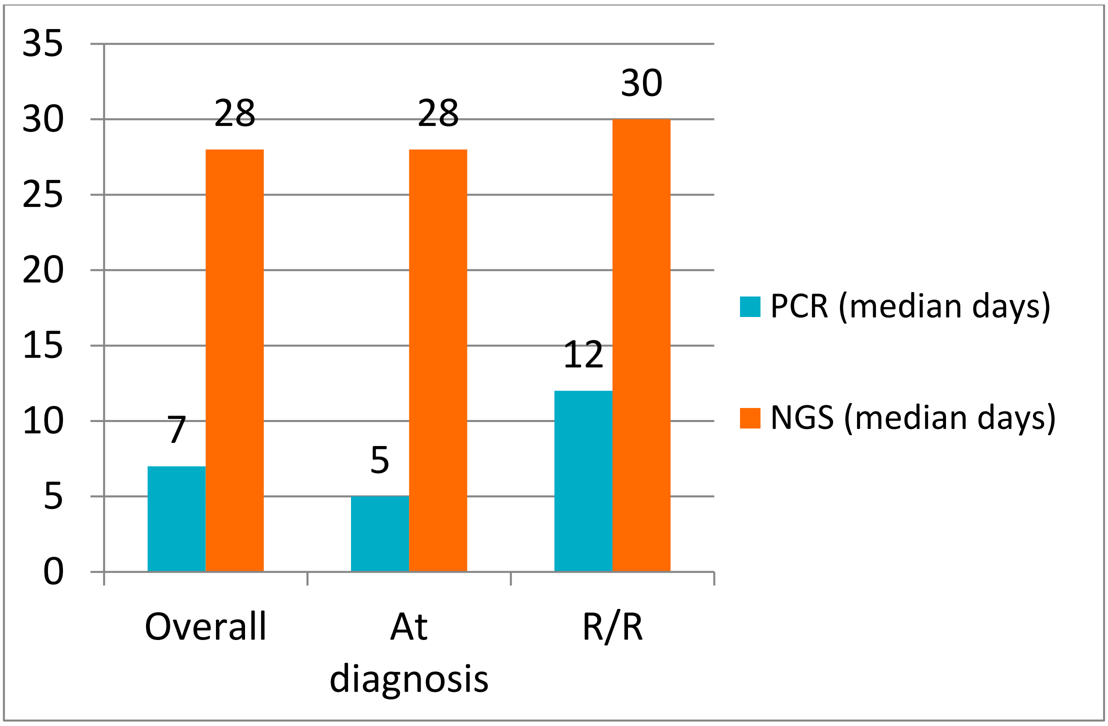

Appendix: Institutions and clinicians participating in the PETHEMA epidemiologic registry of acute myeloid leukemia and acute promyelocytic leukemia.

Argentina (Grupo Argentino para el Tratamiento de la Leucemia Aguda—GATLA)—Hospital de Clínicas, Buenos Aires: F. Rojas; H. Longoni; Fundaleu, Buenos Aires: G. Milone, I. Fernández, Clínica Conciencia, Neuquén: R. Ramirez; Hospital Rossi, La Plata: C. Canepa, S. Saba, G. Balladares, Hospital General San Martin, Parana: G. Milone, C. Ventiurini, R. Mariano, P. Negri; Hospital Italiano de La Plata, La Plata: M. V. Prates, J. Milone; Hospital General San Martín, La Plata:P. Fazio, M. Gelemur; Hospital Clemente Alvarez,

Rosario: G. Milone, S. Ciarlo, F. Bezares; Hospital de Córdoba, Córdoba: L. López, Hospital Privado de Córdoba, Córdoba: J. J.García; Instituto Privado Hematología, Paraná: P. Negri, M. Giunta, G. Milone; Hospital Teodoro Alvarez, Buenos Aires: M. Kruss; Hospital Tornú, Buenos Aires: D. Lafalse, G. Milone; Hospital Gobernador Centeno, La Pampa: E. Marquesoni, M. F. Casale; Hospital Italiano de Buenos Aires, Buenos Aires: A. Gimenez, E. B. Brulc, M. A. Perusini; Complejo Médico Policía Federal, La Plata: G. Milone, L. Palmer; Colombia (Asociación Colombian de Hematología y Oncología—ACHO)—Clínica La Estancia, Popayán: M. E. Correa; Fundación Valle del Lili, Cauca: F.J. Jaramillo, J. Rosales; Instituto FOSCAL, Bucaramanga: C. Sossa, J. C. Herrera; Hospital Pablo Tobón Uribe, Antioquia: M. Arango; Poland (Polish Adult Leukemia Group—PALG)—City Hospital Legnica, Baja Silesia: J. Holojda; IHIT Hematology and transfusiology institute, Warszawa: A. Golos, A. Ejduk; Wojewódzki Szpital Specjalistyczny w Olsztynie, Olsztyn: B. Ochrem; WIM (Military Institute of Medicine in Warsaw), Warszawa: G. Małgorzata; Poland Medical University of Warsaw Banacha, Warszawa:A. Waszczuk-Gajda, J. Drozd-Sokolowska, M. Czemerska, M. Paluszewska; Medical University School Gdansk, Gdansk: E. Zarzycka; Wojewódzki Szpital Specjalistyczny im. Św.. Jadwigi Śląskiej, Opole: A. Masternak; Hospital Brzozow, Brzozow: Dr. Hawrylecka; Medical University Lublin, Lublin: M. Podhoreka, K. Giannopoulos, T. Gromek; Medical University Bialystok, Bialystok: J. Oleksiuk; Silesian Medical University Katowice, Katowice:bA. Armatys, G. Helbig; Universitary Hospital Wroclaw, Wroclaw: M. Sobas; Poznan University of Medical Sciences, Pozna: A. Szczepaniak; Rydigier City Hospital Krakow, Krakow: E. Rzenno, M. Rodzaj; Collegium Medicum Jagiellonian University Krakow, Krakow: B. Piatkowska-Jakubas; City Hospital Rzeszów, Rzeszów: A. Skret; Medical University Lodz, Lodz: A. Pluta, M. Czemerska; Center of Oncology Kielce, Kielce: E. Barańska; Medical University of Warsaw, Warsaw: M. Paluszewska; Portugal—Hospital de Santa Maria-Lisboa, Lisboa:G. Vasconcelos, J. Brioso; IPOFG Lisboa, Lisboa: A. Nunes, I. Bogalho; Centro Hospitalar e Universitário de Coimbra, Coimbra: A. Espadana, M. Coucelo, S. Marini, J. Azevedo, A. I. Crisostomo, L. Ribeiro, V. Pereira; Centro Hospitalar de Lisboa Central E. P. E, Lisboa: A. Botelho; Instituto Português Oncologia do Porto Francisco Gentil, Porto: J. M. Mariz; Centro Hospitalar São João, Porto: J. E. Guimaraes, E. Aguiar; Centro Hospitalar do Porto E.P.E. Porto: J. Coutinho; Spain (Programa Español de Tratamiento de las Hemopatías Malignas, PETHEMA)—Complejo Hospitalario Universitario A Coruña, A Coruña: V. Noriega, L. García, C. Varela, G. Debén, M. R. González; Hospital Clínico Universitario de Santiago, A Coruña: M. Encinas, A. Bendaña, S. González, J.L. Bello, M. Albors; Hospital General de Albacete, Albacete: L. Algarra, J.R.Romero, J.S.Bermon, M.J. Varo; Hospital Vinalopó, Alicante: V. López, E. López; Hospital Virgen de los Lirios, Alcoy: C. Mora, C. Amorós; Hospital General Elche, Alicante: E. López, A. Romero; Hospital Torrevieja Salud, Alicante: A. Jaramillo, N. Valdez, I. Molina, A. Fernández, B. Sánchez; Hospital de la Marina Baixa Villajoyosa, Alicante: A. García; Hospital General de Elda, Alicante: V. Castaño, T. López, J. Bernabeu; Hospital de Denia-Marina Salud, Alicante: M.J. Sánchez; Hospital de la Vega Baja de Orihuela, Alicante: C. Fernández; Hospital General de Alicante, Alicante: C. Gil, C. Botella, P. Fernández, M. Pacheco, F. Tarín; J.J. Verdú; Complejo Hospitalario Torrecardenas, Almeria: M.J. García, A. Mellado, M.C. García, J. González; Hospital Central de Asturias, Asturias: T. Castillo, E. Colado, S. Alonso; Complejo Asistencial Ávila, Ávila: I. Recio, M. Cabezudo, J. Davila, M. J. Rodríguez, A. Barez, B. Díaz; Hospital Don Benito-Villanueva, Badajoz: J. Prieto; Institut Catala d'Oncologia L'Hospitalet, Barcelona: M. Arnan, C. Marín, M. Mansilla; Hospital de Cruces, Bizkaia: A. Balaberdi, M. E. Amutio, R. A. del Orbe, I. Ancin, J. C. Ruíz; Hospital Galdakao-Usansolo, Bizkaia: M. Olivalres, C. Gómez, I. gonzález, M. Celis, K. Atutxa, T. Carrascosa, T. Artola, M. Lizuain; Basurtuko Ospitalea, Bizkaia: J.I. Rodriguez, O. Arce, J. A. Márquez, J. Atuch, F. Marco de Lucas, Z. Díez, B. Dávila; Hospital Santos Reyes, Burgos: R. Cantalejo, M. Díaz; Hospital Universitario de Burgos, Burgos: J. Labrador, F. Serra, G. Hermida, F. J. Díaz, P. de Vicente, R. Álvarez: Hospital Santiago Apóstol, Burgos: C. Alonso, Hospital San Pedro de Alcántara, Cáceres: J. M. Bergua; Hospital Campo Arañuelo, Cáceres: N. Ugalde; Hospital Virgen del Puerto, Cáceres: E. Pardal; Hospital General Jerez de la Frontera, Cádiz: R. Saldaña, F. Rodríguez, E. Martín, L. Hermosín; Hospital Universitario Puerta del Mar, Cádiz: M. P. Garrastazul, I. Marchante, J. A. Raposo, F. J. Capote; Hospital U. Marqués de Valdecilla, Cantabria: M. Colorado, A. Batlle, L. Yañez, S. García, P. González, E. M. Ocio, M. Briz, A. Bermúdez, S. García; Consorcio Hospitalario Provincial de Castellón, Castellón: C. Jiménez, S. Beltrán;

Hospital de Vinaroz: M. Montagud; Hospital Universitario de La Plana, Castellón: I. Castillo; Hospital General de Castellón, Castellon: R. García, A. Gascón, J. Clavel, A. Lancharro, L. Lnares; Hospital Santa Bárbara, Ciudad Real: M. M. Herráez, A. Milena; Hospital Virgen de Altagracia, Ciudad Real: M. J. Romero, Hospital General de Ciudad Real, Ciudad Real: B. Hernández, C. Calle, R. Benegas; Hospital Gutierrez Ortega de Valdepeñas, Ciudad Real: Dr. Bolívar; Hospital General La Mancha Centro, Ciudad Real: M. A. Pozas; Hospital Reina Sofia, Córdoba: J. Serrano, F. J. Dorado, J. Sánchez, M. C. Martínez; Hospital Virgen de la Luz, Cuenca: C. J. Cerveró, M. J. Busto; Hospitales HUVN-HC San Cecilio de Ganada, Granada: M. Bernal, E. López, L. Moratalla, Z. Mesa, M. Jurado, A. Romero, P. González; Complejo Hospitalario Universitario Granada, Granada: L. Moratalla, A. Romero, L. López; Hospital Universitario de Guadalajara, Guadalajara: M. Díaz, D. De Miguel, A. B. Santos, J. Arbeteta; Hospital Donostia, Donosti: E. Pérez, N. Caminos, N. Uresandi, N. Argoitiaituart, T. Artola, J. Swen, A. Uranga, I. Olazaba, M. Lizuain, E. Gainza, P. Romero; Hospital Juan Ramón Jimenez Huelva, Huelva: E. Gil, A. J. Palma, K. G. Gómez, M. Solé, J. N. Rodríguez; Hospital San Jorge, Huesca: I. M. Murillo, J. Marco, J. Serena, V. Marco; Hospital de Barbastro, Huesca: M. Perella, L. Costilla; Hospital General Ciudad de Jaen, Jaén: J. A. López, A. Baena, P. Almagro; Hospital San Pedro de Logroño, La Rioja: M. Hermosilla, A. Esteban, B. A. Campeny, M. J. Nájera, P. Herrra; Hospital Insular de Las Palmas, Las Palmas: R. Fernández, J. D. González, L. Torres; Hospital Dr. Negrín, Las Palmas: S. Jiménez; M. T. Gómez, C. Bilbao, C. Rodríguez; Hospital Doctor José Molina Orosa, Las Palmas: A. Hong, Y. Ramos de Laón, V. Afonso; Hospital Universitario de León, León: F. Ramos, M. Fuertes; Hospital Comarcal del Bierzo, León: E. de Cabo, C. Aguilera, M. Megido; Hospital Universitari Arnau de Vilanova de Lleida, Leida: T. García; Hospital Lucus Augusti, Lugo: E. Lavilla, M. Varela, S. Ferrero, M. J. Sánchez, L. López, J. Arias, L. Vizcaya; Hospital Infanta Sofía, Madrid: A. Roldán, A. Vilches, M. J. Penalva, J. Vázquez; Hospital Central de la Defensa Gómez Ulla, Madrid: M. T. Calderón, A. Matilla, C. Serí, M. J. Otero, N. García, E. Sandoval; Hospital de Fuenlabrada, Madrid: C. Franco, R. Flores, P. Bravo, A. López; Hospital Fundación Jiménez Díaz, Madrid: J. L. López, C. Blas, A. Díez, J. M. Alonso, C. Soto, A. Arenas; Hospital U. Príncipe de Asturias, Madrid: J. García, Y. Martín, P. S. Villafuerte, E. Magro; Hospital Puerta de Hierro, Madrid: G. Bautista; A. De Laiglesia; Hospital Gregorio Marañón, Madrid: G. Rodríguez, L. Solán, M. Chicano, P. Balsalobre, S. Monsalvo, P. Font, D. Carbonell, C. Martínez; Hospital U. La Paz, Madrid: K. Humala, A. E. Kerguelen, D. Hernández, M. Gasior, P. Gómez, I. Sánchez; Hospital Madrid Norte Sanchinarro, Madrid: S. Redondo, L. Llorente, M. Bengochea, J. Pérez; Hospital Sanitas Torrejón, Madrid: A. Sebrango, M. santero, A. Morales; Hospital La Princesa, Madrid: A. Figuera, P. Villafuerte, A. Alegre, E. Fernández; Hospital Ruber Internacional, Madrid: A. Alonso; Hospital 12 de Octubre, Madrid: M. P. Martínez, J. Martínez, M. T. Cedena, L. Moreno; MD Anderson Cancer Center, Madrid: A. De la Fuente; Hospital Sanitas La Zarzuela, Madrid: D. García; Hospital Universitario Quiron, Madrid: C. Chamorro, V. Pradillo, E. Martí, J. M. Sánchez, I. Delgado, A. Alonso; Hospital Rey Juan Carlos, Madrid: B. Rosado, A. Velasco, C. Miranda, G. Salvatierra, J. M. Alonso,, J. L. López; Hospital Infanta Leonor, Madrid: M. Foncillas, J. A. Hernández; Hospital Universitario de Getafe, Madrid: C. Escolano, L. García, I. Delgado; Hospital Clínico San Carlos, Madrid: C. Benabente, R. Martínez, M. Polo, E. Anguita; Hospital Universitario Severo Ochoa, Madrid: R. Riaza, G. Amores, M. J. Requena; Hospital Universitario Fundación Alcorcón, Madrid: F. Javier, L. Villaloón; Hospital Universitario Moncloa, Madrid: C. Aláez, V. Pradillo, S. Nistal, B. Navas; Hospital Universitario de Móstoles, Madrid: J. Sánchez, M. A. Andreu; Hospital Ramon y Cajal, Madrid: P. Herrera, J. López; Hospital U. Virgen de la Victoria, Málaga: M. García, M. J. Moreno, A. Fernández, M. P. Queipo; Hospital Quirónsalud Málaga, Málaga: A. Hernández; Hospital Regional de Málaga, Málaga: M. Barrios, A. Heiniger, A. Jiménez, A. Contento, F. López, M. Alcalá; Hospital Vithas Xanit Internacional, Málaga: S. Lorente, M. González, E. M. Morales, J. Gutierrez; Hospital Virgen del Castillo, Murcia: M. J. Serna, V. Beltrán; Hospital Santa Lucía de Cartagena, Murcia: M. Romera, M. Berenguer, A. Martínez, A. Tejedor; Hospital Morales Meseguer, Murcia: M. L. Amigo, F. Ortuño, L. García, A. Jerez, O. López; Hospital U. Virgen de la Arrixaca, Murcia: J. M. Moraleda, P. Rosique, J. Gómez, M. C. Garay; Hospital Los Arcos Mar Menor, Murcia: P. Cerezuela, C. Martínez, A. B. MArtínez, A. González; Hospital STª Mª del Rosell, Murcia: J. Ibáñez; Clínica San Miguel, Navarra: M. J. Alfaro; Complejo Hospitalario de Navarra, Navarra: M. Mateos, M. A. Goñi, M. A. Araiz, A. Gorosquieta, M. Zudaire, M. Viguria, A. Zabala, M. Alvarellos, I.

Quispe, M. P. Sánchez, G. Hurtado, M. Pérez, Y. Burguete, N. Areizaga, T. Galicia; Clínica Universitaria de Navarra, Navarra: J. Rifón, A. Alfonso, F. Prósper, M. Marcos, L. E. Tamariz, V. Riego. A. Manubens, M. J. Larrayoz, M. J. Calasanz, A. Mañú, B. Paiva, I. Vázquez, L. Burgos; Complejo Hospitalario de Ourense (CHOU), Ourense: M. Pereiro, M. Rodríguez, M. C. Pastoriza, J. A. Mendez, J. L. Sastre, M. Iglesias, C. Ulibarrena, F. Campoy; Hospital Valdeorras, Ourense: D. Jaimes; Hospital Rio Carrión, Palencia: J. M. Alonso, B. Albarrán, J. Solano, A. Silvestre; Complexo Hospitalario Universitario de Vigo, Vigo: C. Albo, S. Suarez, C. Loureiro, I. Figueroa, M. Rodríguez, M. A. Fernández, A. Martínez, C. Poderós, J. Vazquez, L. Iglesias, A. Nieto, T. Torrado, A. M. Martínez; Hospital Provincial de Pontevedra, Pontevedra: M.L. Amador, P. Oubiña, E. Feijó, A. Dios, I. Loyola, R. Roreno; Hospital POVISA, Pontevedra: A. Simiele, L. Álvarez, V. Turcu; Hospital U. Salamanca, Salamanca: B. Vidriales, M. González, R. García, A. Avendaño, C. Chillón, E. Pérez, V. González; Hospital General La Palma, Santa Cruz de Tenerife: J. V. Govantes, S. Rubio, M. Tapia; Hospital General de Segovia, Segovia: C. Olivier, J. A. Queizán; Hospital U. Virgen Macarena, Sevilla: O. Pérez, J. A. Vera, C. Muñoz, A. rodriguez, N. González; Hospital U. Virgen del Rocio, Sevilla: J. A. Pérez, E. Soria, I.Espigado, J. Falantes, I. Montero, P. García, E. Rodríguez, E. Carrillo, T. Caballero, C. García; Hospital Virgen de Valme, Sevilla: C. Couto, I. Simón, M. Gómez; Hospital Virgen del Mirón de Soria, Soria: C. Aguilar; Hospital Universitario Canarias, Tenerife: B. J. González, S. Lakhwani, A. Bienert, B. González; Hospital Universitario Nuestra Señora de Candelaria, Tenrife: A. Cabello, A. Y. Oliva, H. González; Hospital Obispo Polanco, Teruel: N. González, Hospital de Alcañiz, Teruel: L. Sancho, M. Paricio, L. Perdiguer; Hospital General Nuestra Señora del Prado, Toledo: F. Solano, A. Lerma, M. D. Martínez; Hospital Virgen de la Salud de Toledo, Toledo: M. I. Gómez, A. Yeguas; Hospital U. La Fe, Valencia: P. Montesinos, E. Barragán, C. Sargas, R. Amigo, D. Martinez, B. Boluda, R. Rodríguez, E. Acuña, I. Cano; Hospital de Requena, Valencia: A. Escrivá, M. Pedreño; Hospital de Lluis Alcanyis de Xativa, Valencia: R. Renart; IVO (Instituto Valenciano de Oncología), Valencia: A. Navalón; Hospital de Sagunto, Valencia: I. Castillo, M. Orts; Hospital Dr. Peset, Valencia: M. J. Sayas, M. J. Fernández, M. L. Juan, E. Gómez, M. Gimeno, E. Donato, M. Cejalvo, J. Marco; Hospital Clínico Universitario, Valencia: M. Tormo, M. Calabuig, B. Navarro, I. Martin, E. Villamont, A. Miralles; Hospital de La Ribera, Valencia: R. Lluch; Hospital Casa de la Salud, Valencia: J. García; Hospital de Gandía, Valencia: M. Moragues, M. A. Ruiz; Hospital Arnau de Vilanova, Valencia: A. López, C. Benet, M. Valero; Hospital General de Valencia, Valencia: M. Linares, R. Collado, M. Orero, P. Ibañez, M. J. Lis, P. L. Pérez, M. Roig, M. López, A. V. Mena; Hospital Manises, Valencia: I. Picón, V. Cánovas, A. Palacios, E. Martí; Hospital Clínico de Valladolid, Valladolid: R. Cuello, J. Borrego, M. burgois; Hospital Rio Hortega, Valladolid: A. Cantalapiedra, O. Norberto, E. Angomas, B. Cidoncha; Hospital Universitario Araba, Victoria: L. Cuevas, D. Robles, A. Mendiazabal, I. Oiartzabal, J. M. Guinea de Castro; Hospital Virgen de la Concha, Zamora: C. Montes, M. Pérez, L. García; Hospital Royo Villanova, Zaragoza: V. Carrasco, A. Pérez, L. López, J. J. Moneva; Hospital Clínico U. Lozano Blesa, Zaragoza: M. Olave, E. Bonafonte, L. Mayor, G. Azaceta, L. Palomera; Hospital Ernest Lluch Martin, Zaragoza: M. Malo, M. J. Escobar; Hospital Quiron Salud Zaragoza, Zaragoza: J. M. Grasa; Hospital Miguel Servet, Zaragoza: B. De Rueda, A. Aulés, C. Salvador, V. Ansó, A. Iborra, P. Delagado, A. Rubio; Uruguay—Hospital de Clínicas, Montevideo: M. Stevenazzi, I. Alpire, V. Irigoin, L. Díaz, C. Guillermo, R. Guadagna, S. Grille, C. Oliver, M. Boada, V. Vales; Hospital Maciel, Montevideo: A. I. Prado; COMERO (Cooperativa Medica de Rocha), Rocha: A. P. De los Santos.
